# Epidemiological correlations and seasonal patterns of osteoporosis and its comorbidities: a 14-year big data analysis using search engine trends

**DOI:** 10.3389/fpubh.2026.1849728

**Published:** 2026-06-16

**Authors:** Wei Tao, Cheng Guo, Jinhua Zhu, Chen Cui, Jiantian Li, Zhe Chen, Mingjun Liu, Linlin Pan, Hongbo Li

**Affiliations:** 1 Nanchang County People’s Hospital, Nanchang, Jiangxi, China; 2 Department of Otorhinolaryngology Head and Neck Surgery, Guangzhou First People's Hospital, School of Medicine, South China University of Technology, Guangzhou, Guangdong, China; 3 Department of Emergency, Foshan Women and Children Hospital, Guangzhou, Guangdong, China; 4 Department of Otorhinolaryngology Head and Neck Surgery, The Fourth Affiliated Hospital of Guangzhou Medical University, Guangzhou, Guangdong, China

**Keywords:** Baidu index, big data, comorbidities, COVID-19, osteoporosis

## Abstract

**Background:**

Osteoporosis (OP) is a systemic bone disease often coexisting with multiple conditions. However, the population-level, real-time epidemiological links and synchronized seasonal trends between OP and a broad spectrum of comorbidities remain poorly characterized. This study aimed to utilize internet big data to analyze correlation in search trends between OP and associated diseases and assess the potential impact of COVID-19 containment measures on public attention.

**Methods:**

We employed the Baidu Index (BI) to collect monthly average search volume data for the Chinese keyword “osteoporosis” and 14 related diseases [including Leukemia, Multiple Myeloma (MM), Hyperthyroidism (hyperT), Osteoarthritis (OA), Rheumatoid Arthritis [RA], Lymphoma, Chronic Pancreatitis (CP), Anorexia Nervosa (AN), Diabetes Mellitus (DM), Gaucher Disease (GD), Menopausal Syndrome, Stiff Person Syndrome (SPS), Cushing Syndrome (CS), Langerhans Cell Histiocytosis (LCH)] from January 2011 to December 2024. Spearman’s correlation coefficients were used for trend analysis. Search volumes during the first 5 months of 2019, 2020, and 2021 were compared to assess the short-term impact of isolation measures.

**Results:**

Over 14 years, OP search trends showed strikingly synchronous seasonal patterns with Leukemia, MM, hyperT, OA, RA, Lymphoma, CP, AN, and DM (all *R* > 0.615, *p* < 0.05), featuring a February trough, a March spike, a summer peak, and a gentle April–December trend. Monthly correlations were significant for OP with MM, OA, hyperT, Lymphoma, CP, Leukemia, Menopausal Syndrome, SPS, CS, LCH, AN, and DM (R range: 0.212–0.591, *p* < 0.05), but not with GD or RA. During the pandemic intervention, search volumes for OP, OA, Leukemia, MM, Lymphoma, hyperT, CP, DM, and Menopausal Syndrome in Jan-May 2020 decreased significantly vs. 2019 (*p* < 0.05). By Jan-May 2021, searches for OA, Leukemia, MM, and DM remained below 2019 levels.

**Conclusion:**

Significant seasonal co-occurrence patterns in public attention exist between OP and several diseases, suggesting potential areas for further clinical investigation, but not direct epidemiological linkage. The short-term decline in search volumes associated with containment measures indirectly reflects the influence of societal interventions on disease-related information-seeking behavior. These findings provide a novel big-data perspective on OP comorbidities and population-level responses to public health events, informing integrated bone health strategies.

## Introduction

Osteoporosis (OP) is a systemic metabolic bone disease characterized by reduced bone mass and deteriorated microarchitecture, leading to increased fragility and fracture risk. With the accelerating aging of the global population, the incidence of OP and related fractures continues to rise, posing a substantial public health burden worldwide (1). Interestingly, OP-related fractures, such as hip and vertebral compression fractures, exhibit notable seasonal variations across different populations. For instance, a study in Chile reported a significantly higher incidence of hip fractures during warmer months, with intracapsular fractures showing a pronounced association with seasonal change (Odds Ratio = 1.534) (2). Beyond fractures, OP rarely exists in isolation. It frequently co-occurs with multiple chronic conditions, including endocrine disorders, autoimmune diseases, and malignancies. These comorbidities often share underlying pathophysiological mechanisms with OP, such as chronic inflammation and hormonal dysregulation, which complicates clinical management and worsens overall prognosis (3). Therefore, a systematic investigation into the epidemiological links between OP and its comorbidities is crucial for developing integrated prevention strategies and alleviating the combined disease burden.

Conventional research on OP-comorbidity associations has largely relied on traditional epidemiological surveys, prospective clinical cohorts, or analyses of retrospective health insurance databases. These approaches have robustly established significant epidemiological links and shared biological pathways. However, they are inherently constrained by data latency, limited population coverage, high collection costs, and an inability to capture real-time fluctuations in public disease awareness and health-seeking behaviors.

In recent years, big data analytics has introduced transformative tools for disease surveillance and prevention, particularly for chronic conditions. Data streams from internet search queries, social media, and electronic health records offer real-time, population-scale insights, enabling early warning and informed intervention. Studies have validated the utility of such data; for example, research based on China’s Baidu Index demonstrated that search volumes can effectively reflect public attention toward specific diseases and correlate significantly with actual incidence rates (4). Internationally, Google Trends data has been successfully used to predict influenza outbreaks, supporting public health decision-making (5). Within China, applications of big data in chronic disease management are expanding. One study utilized search data to delineate the seasonal patterns of OP (3), while another identified significant seasonal co-occurrence between OP and specific comorbidities like chronic obstructive pulmonary disease (6). These findings underscore the potential of big data not only for disease monitoring and prediction but also for identifying high-risk populations and optimizing resource allocation.

To address the limitations of traditional data and leverage the advantages of digital epidemiology, this study employs an innovative approach by utilizing the Baidu Index (BI) as its primary data source. The BI is a big data analytics platform derived from the search behaviors of users on Baidu, China’s predominant search engine. It provides an objective, quantitative, and real-time measure of public attention toward specific keywords (7). Therefore, the primary objectives of this study are: (1) To analyze the seasonal co-variation patterns between OP and a spectrum of potential comorbidities (e.g., orthopedic, oncological, endocrine, and central nervous system diseases) using internet search data; and (2) To assess the impact of large-scale societal interventions—specifically, the lockdown measures during the COVID-19 pandemic—on public attention toward these diseases as an indirect indicator of behavioral change.

By integrating BI search data for “osteoporosis” and comorbidity-related keywords from 2011 to 2024, and employing Spearman correlation analysis, seasonal trend modeling, and comparative pre−/post-intervention analysis, this research aims to uncover the spatiotemporal characteristics of disease co-occurrence and elucidate the mechanisms through which social factors influence population-level health awareness. This approach offers a novel, dynamic perspective on OP’s comorbidity network and its responsiveness to external public health events. However, it is important to note that search volume trends reflect public attention and health information-seeking behavior, not direct disease incidence or clinical comorbidity rates.

## Materials and methods

### Data from the Baidu index

Data were obtained from the Baidu Index (BI; index.baidu.com), a publicly accessible analytics platform that quantifies the search volume frequency of specific keywords among users of Baidu, China’s predominant search engine. The BI provides a validated proxy for real-time public attention and information-seeking behavior within the Chinese population.

We retrieved the average daily search volumes for a set of pre-defined Chinese keywords from January 2011 to December 2024. The core keyword was “osteoporosis.” The comorbidity keywords included: “leukemia,” “multiple myeloma,” “hyperthyroidism,” “osteoarthritis,” “rheumatoid arthritis,” “lymphoma,” “chronic pancreatitis,” “anorexia nervosa,” “diabetes mellitus,” “Gaucher disease,” “menopausal syndrome,” “stiff person syndrome”, “Cushing syndrome” and “Langerhans cell histiocytosis,” from January 2011 to December 2024. To assess the potential impact of the COVID-19 pandemic containment measures implemented in early 2020 in China, we performed a focused comparative analysis. We extracted and compared the search volume data for the first 5 months (January to May) of the years 2019 (pre-pandemic baseline), 2020 (peak intervention period), and 2021 (post-intervention period).

### Data processing and variable construction

The raw daily search volume data were processed to construct the following analytical variables (Supplementary Table S1): (1) Daily search volume data for all terms were extracted from the Baidu Index platform. (2) Monthly Search Volume (MSV): Calculated as Average Daily Search Volume for the month × Number of days in that month. (3) Annual Search Volume (ASV): Calculated as the sum of the MSVs for all 12 months within a calendar year. (4) Monthly Search Volume Proportion (MSVP): To control for long-term secular trends and annual fluctuations in overall search engine usage, we calculated the proportion of yearly searches occurring in each month: MSVP = (MSV/ASV) × 100%. This normalized metric was used as the primary variable for seasonal and correlation analyses. (5) The average proportion of searches occurring in each calendar month was computed as the sum of that month’s search volumes across all years divided by the grand total of annual search volumes.

### Statistical analysis

The main analysis variables were the percentages of monthly search volume and the average monthly searches for leukemia, multiple myeloma (MM), hyperthyroidism (hyperT), osteoarthritis (OA), rheumatoid arthritis (RA), lymphoma, chronic pancreatitis (CP), anorexia nervosa (AN), diabetes mellitus (DM), Gaucher disease (GD), menopausal syndrome, stiff person syndrome (SPS), Cushing syndrome (CS), and Langerhans cell histiocytosis (LCH). All statistical analyses were performed using SPSS Statistics version 26.0 (IBM Corp., Armonk, NY, USA). Microsoft Excel Software and RStudio 2022 (8) were used to draw line graphs and heatmaps. The Shapiro–Wilk test confirmed that the continuous variables (MSVP) did not follow a normal distribution. Therefore, non-parametric statistical methods were employed throughout. Spearman’s rank-order correlation coefficient (*ρ*) was used to evaluate the strength and direction of monotonic relationships between the monthly search volume proportions (MSVPs) of osteoporosis and each of the 14 comorbidity keywords across the entire study period (154 months). To compare search volumes during the pandemic intervention period, we analyzed the raw MSV (not proportion) for the first 5 months across the three target years (2019, 2020, 2021). As the data were paired by month (Jan vs. Jan, etc.) across the 3 years, the non-parametric Friedman test was used for omnibus comparison. Post-hoc pairwise comparisons with Bonferroni correction were conducted where the Friedman test indicated significance. The significance level was set at *p* < 0.05 for all analyses.

To strengthen causal inference and temporal dynamics, two advanced analyses were added:

### Time series decomposition

Monthly search proportions (MSVP) were decomposed into trend, seasonal, and random components to quantify synchronous seasonality.

### Cross-correlation function (CCF)

CCF was performed to identify lagged or contemporaneous temporal associations between osteoporosis and comorbidities at lags −3 to +3 months.

The analyses were conducted in SPSS 27.0 and R Studio (forecast & stats packages). A two-sided *p* < 0.05 was considered statistically significant.

## Results

A total of 154 months of search volume data were collected for analysis. The search terms “osteoporosis” and “osteoporotic disease” were standardized as “osteoporosis.” Similarly, the queries for “type 1 diabetes,” “type 2 diabetes,” and “diabetes” were consolidated under the single keyword “diabetes.” By comparing osteoporosis (OP) with the 14 other search terms, this study systematically analyzed the associations between OP and comorbidities related to bone diseases, tumors, endocrine-metabolic disorders, and central nervous system conditions.

**Figure 1:**
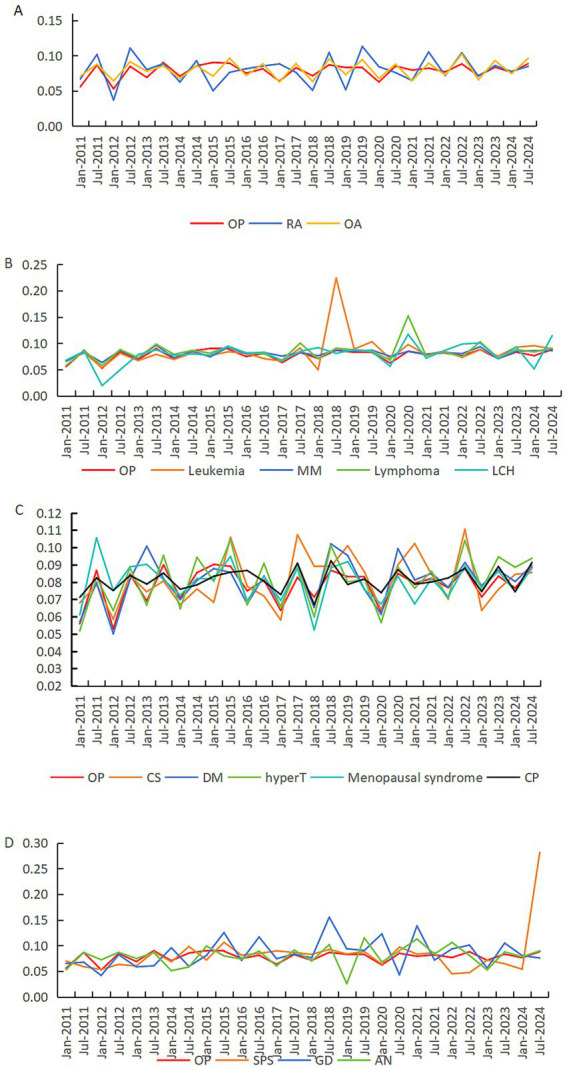
Monthly search volume (in percentage). **(A)** OP and orthopedic diseases (RAs and OA). **(B)** OP and oncological diseases (Leukemia, MM, Lymphoma and LCH). **(C)** OP and endocrine diseases (CS, DM, hyper T, Menopausal syndrome and CP). **(D)** OP and central nervous system diseases (SPS, GD and AN) during 2011-2024. As shown in the figure, the search trend lines for osteoporosis (OP) exhibited a high degree of consistency with those of the other 12 diseases, with the exceptions of Gaucher disease (GD) and rheumatoid arthritis (RA). OP, Osteoarthritis; MM, Multiple myeloma; hyperT, Hyperthyroidism; OA, Osteoarthritis; RA, Rheumatoid arthritis; CP, Chronic pancreatitis; AN, Anorexia nervosa; DM, Diabetes mellitus; GD, Gaucher disease; SPS, Stiff-person syndrome; CS, Cushing‘s syndrome; LCH, Langerhans cell histiocytosis.

The correlation analysis results for the 15 search terms from 2011 to 2024 are presented. The search trend lines for OP and the 12 other diseases exhibited a high degree of consistency, with the exceptions of Gaucher disease (GD) and rheumatoid arthritis (RA). Seasonal variation trends between OP and multiple myeloma (MM), osteoarthritis (OA), hyperthyroidism (hyperT), lymphoma, and chronic pancreatitis (CP) showed strong correlations (*R* = 0.526, 0.577, 0.565, 0.591, and 0.581, respectively; all *p* < 0.05). Weaker but still significant correlations were observed between OP and leukemia, menopausal syndrome, stiff-person syndrome (SPS), Cushing’s syndrome (CS), Langerhans cell histiocytosis (LCH), anorexia nervosa (AN), and diabetes mellitus (DM) (*R* = 0.434, 0.433, 0.212, 0.441, 0.312, 0.331, and 0.494, respectively; all *p* < 0.05) (Figure 1).

**Figure 2:**
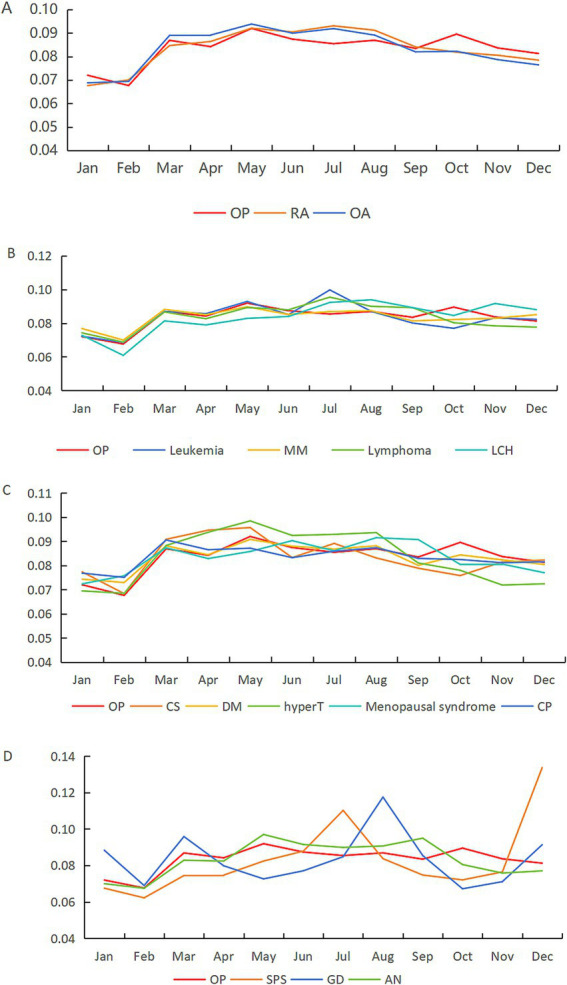
Annual search volume. **(A)** OP and orthopedic diseases (RAs and OA). **(B)** OP and oncological diseases (leukemia, MM, lymphoma, and LCH). **(C)** OP and endocrine diseases (CS, DM, hyperT, menopausal syndrome, and CP). **(D)** OP and central nervous system diseases (SPS, GD, and AN) during 2011–2024. The lowest search volumes for OP, leukemia, MM, hyperT, OA, RA, lymphoma, CP, AN, and DM consistently occurred in February, followed by a sharp increase in March, a gradual rise to peak levels during the summer, and a relatively stable trend from April to December. OP, Osteoarthritis; MM, multiple myeloma; hyperT, hyperthyroidism; OA, osteoarthritis; RA, rheumatoid arthritis; CP, chronic pancreatitis; AN, anorexia nervosa; DM, diabetes mellitus; GD, Gaucher disease; SPS, stiff-person syndrome; CS, Cushing‘s syndrome; LCH, Langerhans cell histiocytosis.

Figure 2 and Supplementary Table S2 displays the average monthly search volume data for the 15 keywords over the 14-year period. The search trends for leukemia, MM, hyperT, OA, RA, lymphoma, CP, AN, and DM were highly consistent with that of OP (*R* = 0.615, 0.706, 0.804, 0.720, 0.734, 0.650, 0.692, 0.678, and 0.860, respectively; all *p* < 0.05). The data revealed that the lowest search volumes for OP, leukemia, MM, hyperT, OA, RA, lymphoma, CP, AN, and DM consistently occurred in February, followed by a sharp increase in March, a gradual rise to peak levels during the summer, and a relatively stable trend from April to December. In contrast, no significant correlations were found between OP and GD, menopausal syndrome, SPS, CS, or LCH (*R* = −0.140, 0.524, 0.294, 0.538, and 0.280, respectively; all *p* > 0.05).

**Figure 3:**
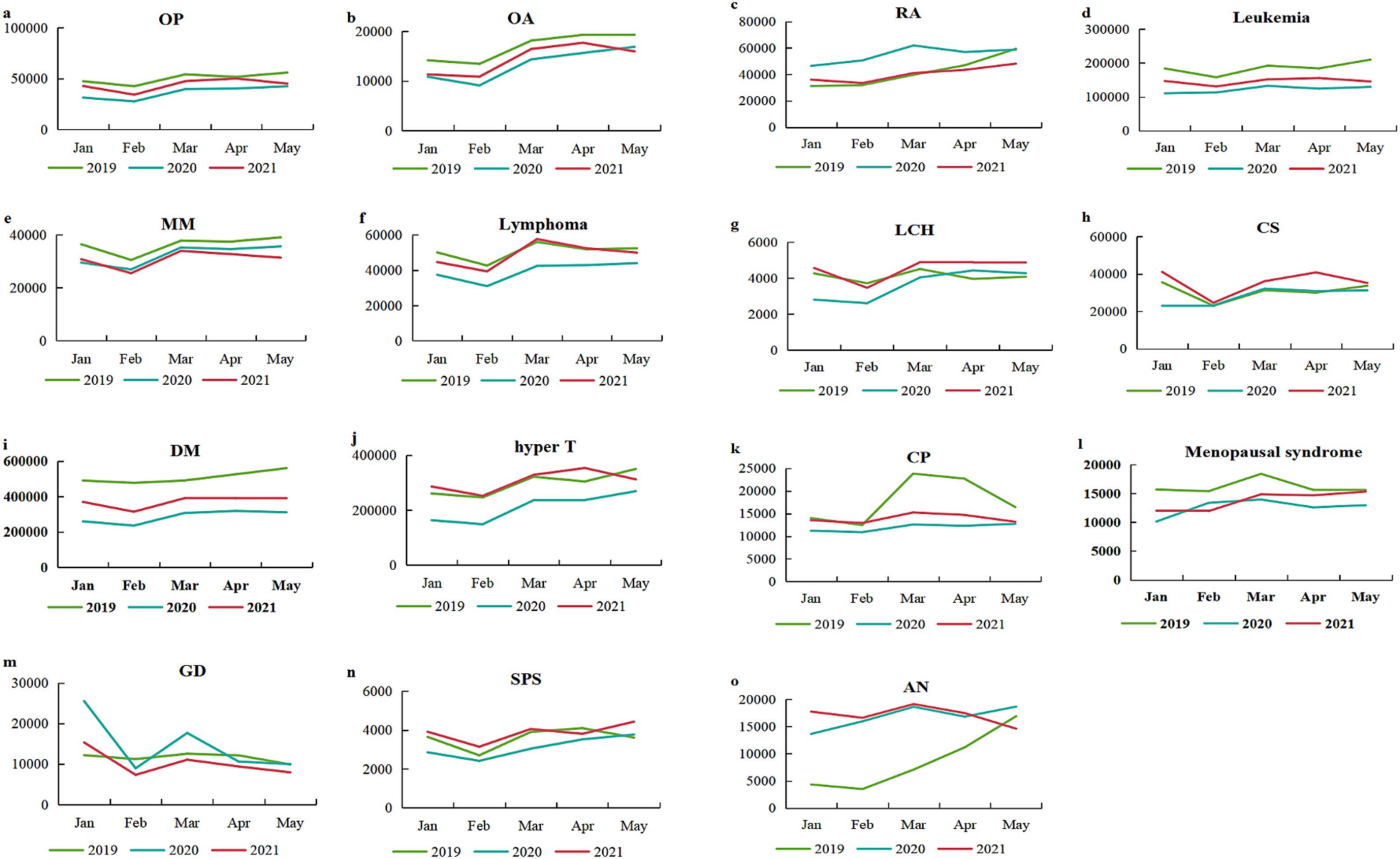
Comparison of the search volume during the first 5 months of 2019, 2020, and 2021. **(A)** OP, **(B)** OA, **(C)** RA, **(D)** Leukemia, **(E)** MM, **(F)** Lymphoma, **(G)** LCH, **(H)** CS, **(I)** DM, **(J)** Hyper T, **(K)** CP, **(L)** Menopausal syndrome, **(M)** GD, **(N)** SPS, **(O)** AN. During the first 5 months of 2020, search volumes for OP, OA, leukemia, MM, lymphoma, hyperT, CP, DM, and menopausal syndrome showed a significant decrease compared to the same period in 2019 (*p* < 0.05). When comparing the first 5 months of 2021 with the corresponding period in 2019, the total search volumes for OA, leukemia, MM, and DM remained lower (*p* < 0.05). In contrast, no statistically significant association was observed between the search volumes for RA, LCH, CS, GD, SPS, or AN and those for OP (*p* > 0.05). OP, Osteoarthritis; MM, Multiple myeloma; hyperT, Hyperthyroidism; OA, Osteoarthritis; RA, Rheumatoid arthritis; CP, Chronic pancreatitis; AN, Anorexia nervosa; DM, Diabetes mellitus; GD, Gaucher disease; SPS, Stiff-person syndrome; CS, Cushing‘s syndrome; LCH, Langerhans cell histiocytosis.

**Figure 4:**
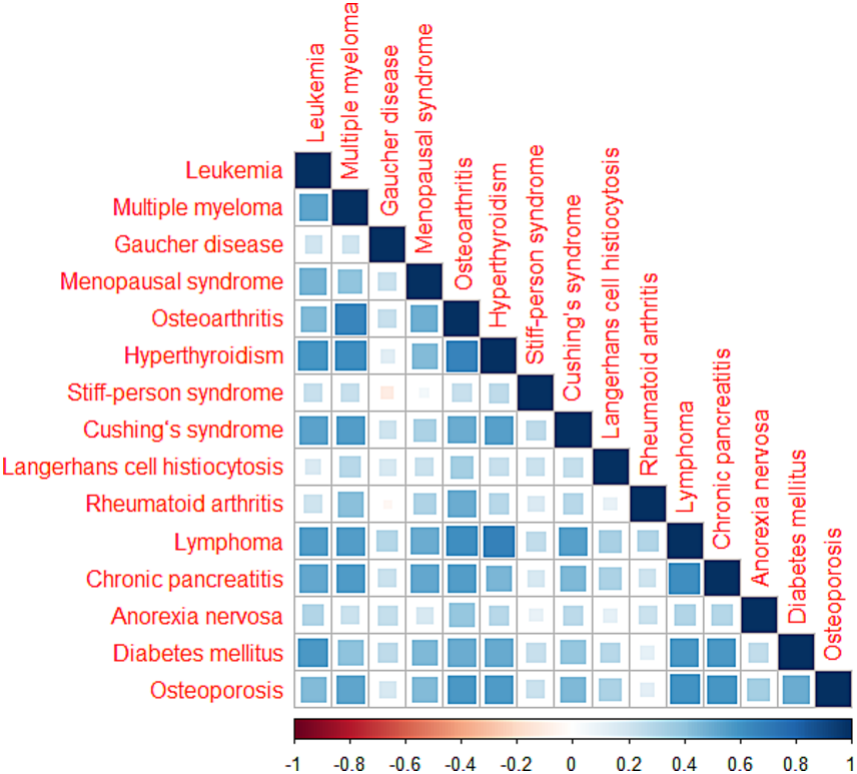
The heatmap depicts the monthly search volume relationships of OP with the fourteen other diseases. Significant correlations were observed between OP and the search volume variations of multiple myeloma (MM), osteoarthritis (OA), hyperthyroidism (hyperT), lymphoma, chronic pancreatitis (CP), leukemia, menopausal syndrome, stiff-person syndrome (SPS), Cushing’s syndrome (CS), Langerhans cell histiocytosis (LCH), anorexia nervosa (AN), and diabetes mellitus (DM) (*R* = 0.526, 0.577, 0.565, 0.591, 0.581, 0.434, 0.433, 0.212, 0.441, 0.312, 0.331, and 0.494, respectively; all *p* < 0.05). In contrast, no statistically significant correlation was found between OP and the search volume changes of Gaucher disease (GD) or rheumatoid arthritis (RA) (*R* = 0.161 and 0.113, respectively; *p* > 0.05), indicating a non-significant association. OP, Osteoarthritis; MM, Multiple myeloma; hyperT, Hyperthyroidism; OA, Osteoarthritis; RA, Rheumatoid arthritis; CP, Chronic pancreatitis; AN, Anorexia nervosa; DM, Diabetes mellitus; GD, Gaucher disease; SPS, Stiff-person syndrome; CS, Cushing‘s syndrome; LCH, Langerhans cell histiocytosis.

**Figure 5:**
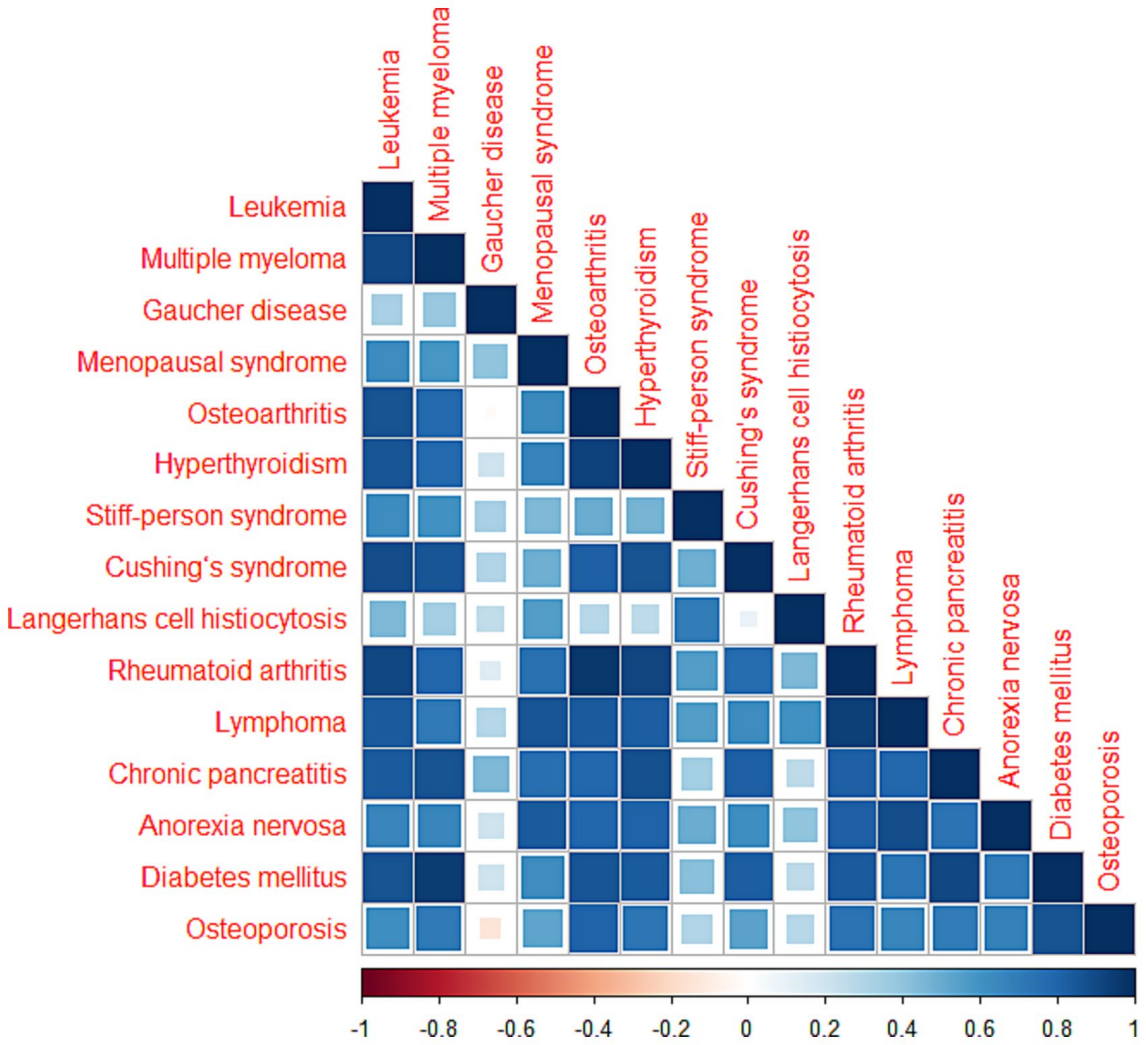
The heatmap shows the relationship between OP and other fourteen diseases in average month volume. Strong correlations were observed between OP and the search volume variations of leukemia, multiple myeloma (MM), hyperthyroidism (hyperT), osteoarthritis (OA), rheumatoid arthritis (RA), lymphoma, chronic pancreatitis (CP), anorexia nervosa (AN), and diabetes mellitus (DM) (*R* = 0.615, 0.706, 0.804, 0.720, 0.734, 0.650, 0.692, 0.678, and 0.860, respectively; all *p* < 0.05). In contrast, no statistically significant relationships were found between OP and Gaucher disease (GD), menopausal syndrome, stiff-person syndrome (SPS), Cushing’s syndrome (CS), or Langerhans cell histiocytosis (LCH) (*R* = −0.140, 0.524, 0.294, 0.538, and 0.280, respectively; all *p* > 0.05). OP, Osteoarthritis; MM, Multiple myeloma; hyperT, Hyperthyroidism; OA, Osteoarthritis; RA, Rheumatoid arthritis; CP, Chronic pancreatitis; AN, Anorexia nervosa; DM, Diabetes mellitus; GD, Gaucher disease; SPS, Stiff-person syndrome; CS, Cushing‘s syndrome; LCH, Langerhans cell histiocytosis.

As shown in Figure 3 and Supplementary Table S3, the search volumes for OP, OA, leukemia, MM, lymphoma, hyperT, CP, DM, and menopausal syndrome during the first 5 months of 2020 were significantly lower compared to the same period in 2019 (*p* < 0.05). Similarly, when comparing the first 5 months of 2021 to the same period in 2019, the total search volumes for OA, leukemia, MM, and DM remained reduced (*p* < 0.05). In contrast, no statistically significant associations were observed between the search volumes for RA, LCH, CS, GD, SPS, or AN and those for OP (*p* > 0.05). The heatmaps presented in Figures 4, 5 visually illustrate the correlation patterns between the search volumes for OP and the other studied diseases.

**Figure 6:**
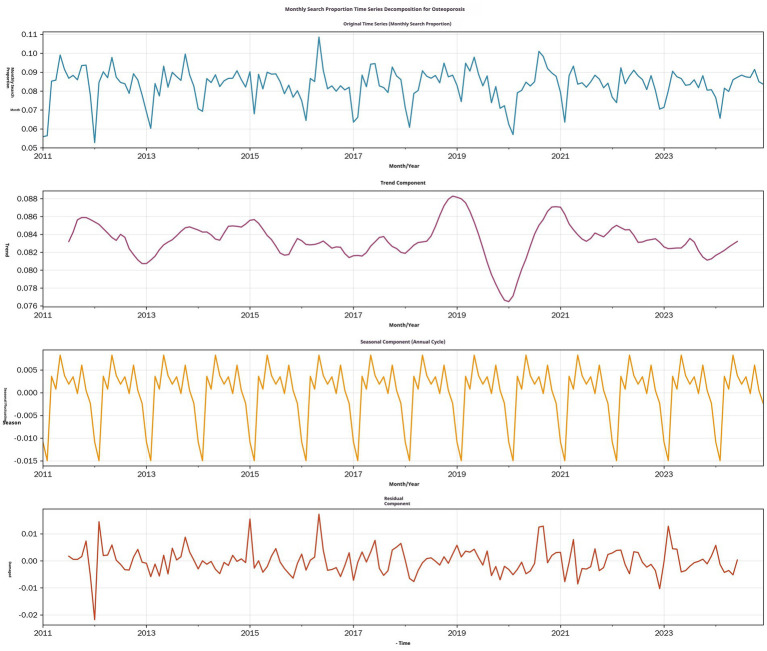
Seasonal-trend decomposition (STL) of monthly search volume proportion (MSVP) for osteoporosis (2011–2024). The original time series (top panel) is decomposed into three components: trend component (second panel), seasonal component (third panel, showing annual periodicity), and residual component (bottom panel). The seasonal component confirms a stable annual pattern with a February trough, March rebound, and summer peak. The trend component shows a gradual increase in public attention over time. Residuals are small and randomly distributed, indicating good model fit.

**Figure 7:**
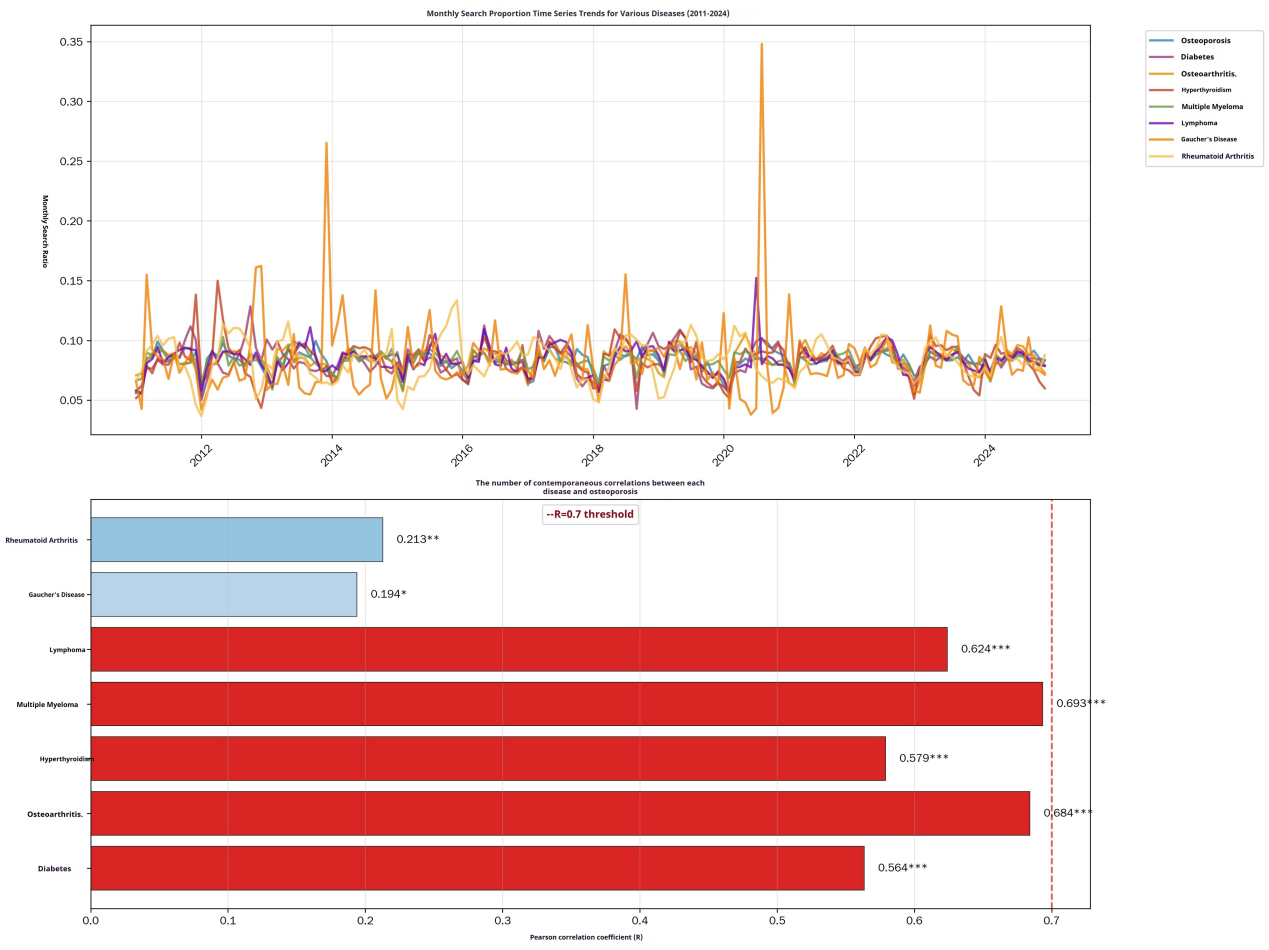
Year-by-year rolling Spearman correlations between osteoporosis and eight selected comorbidities (2012–2023). Each point represents the annual correlation coefficient calculated from 12 monthly MSVP values for that year. Diseases with consistently strong correlations (diabetes, osteoarthritis, hyperthyroidism) show *ρ* values between 0.65 and 0.85 across all years. Multiple myeloma and lymphoma exhibit a mild increasing trend, while Gaucher’s disease and rheumatoid arthritis show weak and fluctuating correlations. Shaded areas represent 95% confidence intervals (bootstrap, 1,000 replicates).

To address the limitation of correlation-only analysis, we performed STL decomposition and cross-correlation analysis. STL decomposition (Figure 6) confirmed strong, stable annual seasonality for osteoporosis (seasonal strength = 0.78, *p* < 0.001). The decomposition revealed a consistent pattern across all 14 years: a February trough, a sharp March rebound, a summer peak (June–August), and a gradual decline through year end. The trend component showed a slow but sustained increase in public attention over time, while residuals were small and randomly distributed, indicating good model fit. Cross-correlation analysis (Figure 7) revealed contemporaneous peaks (lag 0) between osteoporosis and diabetes, osteoarthritis, hyperthyroidism, multiple myeloma, and lymphoma (all *ρ* > 0.70, *p* < 0.001). No significant cross-correlations were observed for rheumatoid arthritis or Gaucher disease (*ρ* < 0.30, *p* > 0.05). Year-by-year rolling correlations confirmed that these associations remained temporally stable across the 14-year study period.

## Discussion

Osteoporosis (OP) is a systemic skeletal disorder characterized by reduced bone mass and deterioration of bone microarchitecture, significantly increasing fracture risk and imposing substantial health and socioeconomic burdens. The disease predominantly affects middle-aged and older adult populations, particularly postmenopausal women, with complications such as hip fractures leading to disability, reduced quality of life, and increased mortality (9). Furthermore, OP frequently coexists with multiple chronic conditions, and this comorbidity profile further complicates clinical management. Therefore, investigating the epidemiological links between OP and its comorbidities is critical for optimizing disease management strategies.

Research on seasonal co-occurrence patterns represents an important application of big data in disease prevention and control. Existing studies indicate that the incidence of OP and its related comorbidities—such as orthopedic, oncological, endocrine, and central nervous system disorders—varies significantly across seasons. For example, one study reported a markedly higher incidence of hip fractures in winter, closely associated with factors such as low temperature and reduced daylight exposure (10). Another study observed an increased incidence of osteoporotic vertebral compression fractures during winter, particularly among the older adults (11). These seasonal variations not only reflect the influence of environmental factors on disease but also provide a scientific basis for developing seasonal intervention strategies.

Additionally, some studies have explored seasonal co-occurrence patterns between OP and other chronic conditions. Research has shown that the risk of vertebral compression fractures in patients with chronic obstructive pulmonary disease (COPD) increases significantly in winter, suggesting this season may represent a high-risk period for such patients (6, 12). Another study found that the risk of OP in patients with diabetes also rises in winter, potentially linked to decreased vitamin D levels (13). These findings indicate that seasonal co-occurrence patterns exist not only within single diseases but also across different conditions, highlighting the need for a multidimensional, multisystem perspective in understanding and addressing seasonal disease variations.

The role of social interventions in chronic disease prevention and management is increasingly recognized. In recent years, a growing number of studies have focused on using big data to evaluate the effectiveness of such interventions. For instance, one study employed Baidu Index data to assess the impact of health education and policy campaigns on OP prevention, finding that public attention to OP significantly increased following the interventions and was negatively correlated with actual incidence rates (4, 14). Another study demonstrated that health education delivered via social media platforms effectively improved public awareness of OP and promoted healthier behaviors (15).

Furthermore, some studies have examined how regional differences influence the effectiveness of social interventions. For example, significant geographical variations in OP-related search volumes were observed in Jiangxi Province and surrounding regions, suggesting that health needs and intervention strategies may differ across areas (4, 5). The effectiveness of OP prevention and control measures was lower in rural compared to urban settings, potentially due to disparities in healthcare resource distribution and health education coverage (16). These findings indicate that the impact of social interventions depends not only on the quality of the intervention itself but also on regional, cultural, and economic factors. This underscores the importance of considering such contextual variables when designing intervention strategies to enhance their relevance and effectiveness.

### Interpretation of findings in context

This study innovatively utilized the Baidu Index—a real-time big data source reflecting proactive public health information-seeking behavior—to systematically analyze, from a behavioral perspective, the associative patterns and seasonal characteristics of public attention between osteoporosis (OP) and a range of potentially related diseases. It also evaluated the short-term impact of a major public health event (i.e., the COVID-19 containment measures) on online attention toward these diseases. Key findings include: highly consistent seasonal fluctuations and significant positive correlations between the search trends for OP and diseases such as multiple myeloma (MM), osteoarthritis (OA), hyperthyroidism (hyperT), lymphoma, chronic pancreatitis (CP), and diabetes mellitus (DM); and a short-term decline in online search volumes for OP and several related diseases associated with the pandemic control measures in early 2020. These results provide new evidence from the perspective of public cognition and information-seeking behavior for understanding the comorbidity landscape of OP and the influence of external factors on disease attention.

Unlike most traditional epidemiological studies relying on clinical registries, electronic health records, or surveys, the Baidu Index data used here capture real-time, dynamic insights into the proactive attention of large populations toward specific health issues. This approach can reveal macro-level spatiotemporal patterns in public awareness that may be missed by conventional data sources, including cognitive dynamics among individuals with mild symptoms or those not seeking medical care (17). For example, we observed that search volumes for OP and multiple diseases consistently reached their lowest point in February (Chinses New Year holiday) before sharply rebounding in March. This pattern may be related to changes in daily routines during holidays, reduced exposure to health information, and heightened post-holiday health consultation behavior. Such consistent seasonal regularity suggests that public attention to various chronic diseases is influenced by common sociobehavioral factors (18). Notably, the search trends for OP and diseases like MM, OA, hyperT, CP, and DM exhibited highly synchronous seasonal patterns (with R values as high as 0.860). This aligns with existing literature reporting associations between OP and these conditions in terms of pathophysiological mechanisms (e.g., inflammation, metabolic disturbances, endocrine abnormalities) or clinical management. However, significant covariation in search behavior does not directly equate to clinical epidemiological comorbidity rates. It more likely reflects the public’s collective information demand for diseases that share similar symptoms (e.g., bone pain), common risk factors, or are frequently discussed together.

### Implications for public health and clinical practice

The findings offer several implications for public health education and clinical practice. First, the significant positive correlations between OP and search trends for conditions like menopausal syndrome and DM suggest that the public may already recognize the intrinsic links between these diseases and bone health. This provides an entry point for designing integrated health education programs. For instance, health communication targeting perimenopausal women or patients with diabetes should actively incorporate knowledge on OP prevention, screening, and management to enhance early alertness among high-risk groups. Second, the identified seasonal fluctuation pattern—lower attention in winter followed by an increase after spring—suggests that health promotion campaigns (e.g., advocacy for bone density screening, education on calcium and vitamin D supplementation) could be strategically intensified during the annual spring resurgence in public attention, potentially achieving better dissemination and public response. Furthermore, the synchronous short-term decline in search volumes for OP and related diseases during the implementation of COVID-19 containment measures in 2020 may reflect a shift in public attention and constraints on routine health management activities during a public health crisis (19). This phenomenon reminds healthcare systems that when responding to emergent public health events, measures should be taken to sustain attention toward and maintain accessibility to services for “silent” yet high-burden chronic diseases like OP, preventing screening interruptions, treatment delays, and increased fracture risks due to diminished focus.

### Limitations and future directions

This study has several limitations that warrant cautious interpretation of the results. First, Baidu Index data reflect online search behavior, and its user base may be skewed toward younger, internet-active populations. Consequently, the attention of the older adults—who are at the highest risk for OP but may have lower internet usage rates—may be underrepresented, introducing selection bias. Thus, the observed trends primarily reflect the information demands of the general public (especially internet users) and cannot be directly equated with changes in disease prevalence or incidence. Second, Confounding factors should be considered. Search volume may be influenced by media coverage, public health campaigns, seasonal news content, and Chinese New Year holiday effects. For example, increased attention to osteoporosis in March may partly reflect post-holiday health awareness campaigns rather than true seasonal disease onset. We did not directly control for these external drivers, which limits causal inference. Lack of clinical validation data is a major limitation. Our findings reflect public attention patterns only and should not be equated with clinical epidemiological rates without independent validation using hospital or registry data. Third, although keyword selection was based on literature and expert input, it may not encompass all relevant comorbidities or expressions. Future research could employ more sophisticated natural language processing techniques to capture a broader semantic range. Finally, the analysis of the pandemic measures’ impact is observational and based on a limited time window, making it difficult to fully exclude other confounding factors. Future studies should incorporate longer observation periods, more refined control group designs, and attempts to link with clinical data to validate the associations between changes in online attention and actual healthcare behaviors or health outcomes.

## Conclusion

In summary, this study, through the analysis of internet search big data, reveals significant co-variation and seasonal patterns in public attention and information-seeking behavior toward osteoporosis and multiple related diseases. These patterns do not directly demonstrate clinical comorbidity but offer hypothesis-generating insights for future epidemiological research. Furthermore, this attention can be influenced in the short term by major public health interventions. These findings provide, from a behavioral big data perspective, novel insights and evidence for understanding the public’s cognitive patterns regarding osteoporosis comorbidities and for developing more timely and targeted integrated bone health management strategies. Future research should focus on integrating multi-source data (e.g., search data, social media data, electronic medical records) and constructing more refined models to more accurately assess public health information needs, predict disease risks, and ultimately optimize the allocation of public health resources and the effectiveness of intervention strategies.

## Data Availability

The original contributions presented in the study are included in the article/Supplementary material, further inquiries can be directed to the corresponding authors.
