# Supplementary material for: Epidemiological correlations and seasonal patterns of osteoporosis and its comorbidities: a 14-year big data analysis using search engine trends
Source: Front Public Health. 2026 Jun 16;14:1849728. doi: 10.3389/fpubh.2026.1849728 (PMC13315008; doi:10.3389/fpubh.2026.1849728)
Supplement: Supplementary file 1 [file Table_1.DOCX]

Supplementary Table S1. Variables and calculations

| Variable | Symbol | Formula of calculation | Example () |
| --- | --- | --- | --- |
| average daily search volume | *D* | Original data from the Baidu Index | *D* (February 2024) = 1196 |
| the number of days in the month | *n* | - | *n* (February) = 29 |
| Monthly Search Volume (MSV) | *M* | *M = D*n* | *M* (February 2024) = 1196*29=34684 |
| Annual Search Volume (ASV) | *Y* | $Y\left( \mathrm{year} \right)=\sum_{i=\mathrm{January}}^{\mathrm{December}} M(i,\mathrm{year})$ | *Y* (2024) = *M* (January 2024) + *M* (February 2024) +…+ *M* (December 2024) = 528550 |
| Monthly Search Volume Proportion (MSVP) | *P* | $P\left( month year \right)=\frac{M(month year)}{Y(\mathrm{year})}$ | *P* (February 2024) = *M* (February 2024) / *Y* (2024) = 34684/528550 = 0.06562 |
| The percentages of average monthly search volume | *P_m_* | $P_{m}=\frac{\sum_{i=2011}^{2024} M(\mathrm{February} i)}{\sum_{i=2011}^{2024} Y(i)}$ | *P_m_* (February) = [*M* (February 2011) + *M* (February 2012) +…+ *M* (February 2021)] / [*Y* (2011) + *Y* (2012) +…+ *Y* (2024)] = 0.0676 |

1. The variable from Baidu index: **average daily search volume (*D*)** of every term.
2. **Monthly Search Volume (MSV):** Calculated as Average Daily Search Volume for the month × Number of days in that month (n).(e.g. The average daily search volume of February 2024 is 1196 times, then the monthly search volume in February 2024 equals 34684 times (1196*29=34684).
3. **Annual Search Volume (ASV):** Calculated as the sum of the MSVs for all 12 months within a calendar year

Results are presented as percentages:

1. **Monthly Search Volume Proportion (MSVP):**  we calculated the proportion of yearly searches occurring in each month: MSVP = (MSV / ASV) × 100%.
2. **The percentages of average monthly search volume** **(*P_m_*)** are obtained by summing the monthly search volumes for the same month throughout the year and dividing the result by the total annual search volume.
