# Supplementary material for: Epidemiological correlations and seasonal patterns of osteoporosis and its comorbidities: a 14-year big data analysis using search engine trends
Source: Front Public Health. 2026 Jun 16;14:1849728. doi: 10.3389/fpubh.2026.1849728 (PMC13315008; doi:10.3389/fpubh.2026.1849728)
Supplement: Supplementary file 2 [file Table_2.DOCX]

Supplementary Table S2. Average monthly search volume

| MONTH | Leukemia | MM | GD | Menopausal syndrome | OA | hyperT | SPS | CS | LCH | RA | Lymphoma | CP | AN | DM | OP |
| --- | --- | --- | --- | --- | --- | --- | --- | --- | --- | --- | --- | --- | --- | --- | --- |
| Jan | 0.0722 | 0.0767 | 0.0883 | 0.0723 | 0.0688 | 0.0694 | 0.0675 | 0.0773 | 0.0728 | 0.0676 | 0.0742 | 0.0768 | 0.0700 | 0.0743 | 0.0720 |
| Feb | 0.0685 | 0.0699 | 0.0690 | 0.0757 | 0.0694 | 0.0684 | 0.0622 | 0.0683 | 0.0608 | 0.0700 | 0.0687 | 0.0750 | 0.0675 | 0.0729 | 0.0676 |
| Mar | 0.0869 | 0.0881 | 0.0958 | 0.0873 | 0.0890 | 0.0882 | 0.0744 | 0.0908 | 0.0813 | 0.0846 | 0.0866 | 0.0905 | 0.0829 | 0.0881 | 0.0869 |
| Apr | 0.0856 | 0.0850 | 0.0798 | 0.0828 | 0.0891 | 0.0937 | 0.0747 | 0.0945 | 0.0789 | 0.0864 | 0.0826 | 0.0865 | 0.0824 | 0.0844 | 0.0842 |
| May | 0.0928 | 0.0897 | 0.0726 | 0.0857 | 0.0938 | 0.0984 | 0.0824 | 0.0956 | 0.0828 | 0.0921 | 0.0893 | 0.0871 | 0.0970 | 0.0907 | 0.0919 |
| Jun | 0.0850 | 0.0851 | 0.0771 | 0.0902 | 0.0899 | 0.0924 | 0.0878 | 0.0832 | 0.0839 | 0.0904 | 0.0879 | 0.0832 | 0.0915 | 0.0879 | 0.0873 |
| Jul | 0.0997 | 0.0868 | 0.0848 | 0.0860 | 0.0918 | 0.0928 | 0.1102 | 0.0891 | 0.0923 | 0.0930 | 0.0954 | 0.0858 | 0.0899 | 0.0867 | 0.0854 |
| Aug | 0.0870 | 0.0874 | 0.1175 | 0.0914 | 0.0891 | 0.0935 | 0.0837 | 0.0831 | 0.0939 | 0.0911 | 0.0900 | 0.0873 | 0.0907 | 0.0881 | 0.0869 |
| Sep | 0.0801 | 0.0813 | 0.0854 | 0.0907 | 0.0819 | 0.0810 | 0.0747 | 0.0788 | 0.0892 | 0.0841 | 0.0892 | 0.0829 | 0.0950 | 0.0800 | 0.0834 |
| Oct | 0.0769 | 0.0821 | 0.0672 | 0.0804 | 0.0822 | 0.0780 | 0.0721 | 0.0758 | 0.0845 | 0.0818 | 0.0802 | 0.0824 | 0.0805 | 0.0843 | 0.0895 |
| Nov | 0.0831 | 0.0828 | 0.0711 | 0.0805 | 0.0786 | 0.0719 | 0.0764 | 0.0814 | 0.0916 | 0.0805 | 0.0784 | 0.0811 | 0.0758 | 0.0823 | 0.0836 |
| Dec | 0.0822 | 0.0850 | 0.0914 | 0.0770 | 0.0764 | 0.0724 | 0.1337 | 0.0822 | 0.0880 | 0.0784 | 0.0776 | 0.0815 | 0.0770 | 0.0804 | 0.0812 |
