# Supplementary material for: Epidemiological correlations and seasonal patterns of osteoporosis and its comorbidities: a 14-year big data analysis using search engine trends
Source: Front Public Health. 2026 Jun 16;14:1849728. doi: 10.3389/fpubh.2026.1849728 (PMC13315008; doi:10.3389/fpubh.2026.1849728)
Supplement: Supplementary file 3 [file Table_3.DOCX]

Supplementary Table S3. The search volume during the first 5 months from 2019 to 2021

|  | Year | Jan | Feb | Mar | Apr | May |
| --- | --- | --- | --- | --- | --- | --- |
| OP | 2019 | 47554 | 42616 | 54219 | 51840 | 56048 |
|  | 2020 | 31403 | 27664 | 39773 | 40410 | 42594 |
|  | 2021 | 42811 | 34300 | 47585 | 50280 | 45136 |
| Leukemia | 2019 | 183781 | 157773 | 192150 | 183976 | 209932 |
|  | 2020 | 110517 | 112949 | 132500 | 124181 | 129492 |
|  | 2021 | 146824 | 130633 | 151727 | 155353 | 145435 |
| MM | 2019 | 36401 | 30473 | 37770 | 37411 | 39010 |
|  | 2020 | 29465 | 26887 | 35185 | 34567 | 35616 |
|  | 2021 | 30739 | 25484 | 33907 | 32687 | 31324 |
| GD | 2019 | 12181 | 11205 | 12529 | 12131 | 9880 |
|  | 2020 | 25517 | 8936 | 17655 | 10585 | 9988 |
|  | 2021 | 15311 | 7315 | 11061 | 9378 | 7933 |
| Menopausal syndrome | 2019 | 15665 | 15383 | 18385 | 15594 | 15566 |
|  | 2020 | 10122 | 13367 | 13936 | 12580 | 12956 |
|  | 2021 | 11985 | 11935 | 14840 | 14662 | 15310 |
| OA | 2019 | 14143 | 13422 | 18101 | 19265 | 19250 |
|  | 2020 | 10822 | 9088 | 14334 | 15619 | 16860 |
|  | 2021 | 11286 | 10856 | 16412 | 17658 | 15946 |
| hyperT | 2019 | 260224 | 246011 | 321276 | 303830 | 349623 |
|  | 2020 | 163080 | 148478 | 236051 | 236324 | 268668 |
|  | 2021 | 285601 | 251617 | 328090 | 353010 | 311727 |
| SPS | 2019 | 3639 | 2693 | 3896 | 4090 | 3605 |
|  | 2020 | 2849 | 2410 | 3035 | 3512 | 3761 |
|  | 2021 | 3903 | 3135 | 4047 | 3799 | 4422 |
| CS | 2019 | 35544 | 23004 | 31305 | 29964 | 33704 |
|  | 2020 | 22997 | 23195 | 32163 | 30844 | 31224 |
|  | 2021 | 41109 | 24579 | 36144 | 40832 | 35200 |
| LCH | 2019 | 4257 | 3710 | 4501 | 3953 | 4070 |
|  | 2020 | 2797 | 2601 | 4032 | 4418 | 4265 |
|  | 2021 | 4556 | 3456 | 4879 | 4863 | 4851 |
| RA | 2019 | 31142 | 31924 | 39684 | 46901 | 59379 |
|  | 2020 | 46420 | 50573 | 61870 | 56944 | 58763 |
|  | 2021 | 35982 | 33418 | 41005 | 43400 | 48117 |
| Lymphoma | 2019 | 50000 | 42530 | 55910 | 51731 | 52315 |
|  | 2020 | 37324 | 30881 | 42302 | 42801 | 43902 |
|  | 2021 | 44514 | 39250 | 57529 | 52303 | 49875 |
| CP | 2019 | 13995 | 12451 | 23822 | 22716 | 16434 |
|  | 2020 | 11222 | 10894 | 12588 | 12295 | 12742 |
|  | 2021 | 13535 | 12908 | 15241 | 14704 | 13182 |
| AN | 2019 | 4325 | 3492 | 7073 | 11185 | 16900 |
|  | 2020 | 13644 | 15968 | 18632 | 16825 | 18658 |
|  | 2021 | 17736 | 16608 | 19124 | 17461 | 14608 |
| DM | 2019 | 490363 | 476957 | 490688 | 525706 | 560327 |
|  | 2020 | 259675 | 235505 | 307189 | 318220 | 310957 |
|  | 2021 | 369498 | 313964 | 391050 | 390837 | 392469 |
